# *Fusarium* Species in Mangrove Soil in Northern Peninsular Malaysia and the Soil Physico-Chemical Properties

**DOI:** 10.3390/microorganisms9030497

**Published:** 2021-02-26

**Authors:** Wafa S. Mohamed Zubi, Masratul Hawa Mohd, Nik Mohd Izham Mohamed Nor, Latiffah Zakaria

**Affiliations:** School of Biological Sciences, Universiti Sains Malaysia, Penang 11800, USM, Malaysia; wwwafaaa@yahoo.com (W.S.M.Z.); masratulhawa@usm.my (M.H.M.); nikizham@usm.my (N.M.I.M.N.)

**Keywords:** *Fusarium* species, FSSC, mangrove soil, phylogenetic, soil analysis, *TEF-1α*

## Abstract

*Fusarium* genus comprises important saprophytic and phytopathogenic fungi and is widespread in nature. The present study reports the occurrence of *Fusarium* spp. in soils from two mangrove forests in northern Peninsular Malaysia and analyzed physico-chemical properties of the mangrove soil. Based on TEF-1α sequences, nine *Fusarium* species were identified: *Fusarium solani* species complex (FSSC) (*n* = 77), *Fusarium verticillioides* (*n* = 20), *Fusarium incarnatum* (*n* = 10), *Fusarium proliferatum* (*n* = 7), *Fusarium lateritium* (*n* = 4), *Fusarium oxysporum* (*n* = 3), *Fusarium rigidiuscula* (*n* = 2), *Fusarium chlamydosporum* (*n* = 1)*,* and *Fusarium camptoceras* (*n* = 1); FSSC isolates were the most prevalent. Phylogenetic analysis of the combined TEF-1α and ITS sequences revealed diverse phylogenetic affinities among the FSSC isolates and potentially new phylogenetic clades of FSSC. Soil analysis showed varied carbon content, pH, soil moisture, and salinity, but not nitrogen content, between sampling locations. Regardless of the physico-chemical properties, various *Fusarium* species were recovered from the mangrove soils. These were likely saprophytes; however, some were well-known plant pathogens and opportunistic human pathogens. Thus, mangrove soils might serve as inoculum sources for plant and human pathogenic *Fusarium* species. The present study demonstrates the occurrence of various *Fusarium* species in the extreme environment of mangrove soil, thereby contributing to the knowledge on species diversity in *Fusarium*.

## 1. Introduction

The genus *Fusarium* is a large taxonomically complex genus comprising more than 200 species [1]. Most *Fusarium* species are species complexes that appear morphologically similar but are genetically closely related and form species complexes, such as the *Fusarium solani* species complex (FSSC) and the *Fusarium fujikuroi* species complex (FFSC). Many species within the species complex are cosmopolitan, distributed in the tropics and temperate regions, and are important plant pathogens as well as opportunistic human pathogens.

As a plant pathogen, *Fusarium* caused various types of diseases including wilt, stem rot, fruit rot, root rot, blight, and cankers in diverse crops such as industrial crops, fruit crops, legumes, ornamentals, and cereal crops. The ability of *Fusarium* to infect various crops is associated with the capability of the fungus to occupy a wide range of substrates, various modes of survival and their cosmopolitan occurrence in agricultural and natural ecosystems [2].

A number of plant pathogenic *Fusarium* spp. produced mycotoxins in the field or during storage. Among well-known mycotoxins produced by mycotoxigenic *Fusarium* are fumonisins, zearalenones, trichothecenes, deoxynivalenol, and moniliformin. These mycotoxins contaminated food and feed which in turn can cause harmful effects on humans and animals. Therefore, plant pathogenic and mycotoxigenic *Fusarium* spp. have the ability to reduce crop yield as well as reduce the quality of agricultural products. In terms of global significance, *Fusarium* has serious implications on socio-economic and international agriculture trade [3].

One of the important soil inhabitants is *Fusarium*, comprising both pathogenic and non-pathogenic strains associated with plants, animals, and humans. Many *Fusarium* species are soil-borne pathogens and facultative parasites. As soil-borne pathogens, *Fusarium* species have been reported in several agricultural or cultivated soils, and the presence of *Fusarium* is commonly associated with plant debris and plant roots. *Fusarium* species have also been isolated from non-agricultural soils, such as peat soil [4], arid environment [5], and Arctic soil [6] which are considered extreme environments.

Microflora in mangrove soils comprises a combination of terrestrial, marine, and freshwater microorganisms [7]. The mangrove soil microbial community plays a major role in nutrient transformation, from residual dead mangrove plants to nitrogen, phosphorus, and other nutrients. Manglicolous fungi are mangrove fungi that are important in nutrient cycling in mangrove soil habitats [8,9] as they facilitate the decomposition of mangrove materials [10].

In Malaysia, although studies on fungal communities in mangrove ecosystems have been conducted, they focused on marine fungi and phylloplane fungi [11,12]. Studies on soil microfungi in mangrove ecosystems are still lacking, even though these types of fungi are vital as decomposers for ecological function. *Fusarium* is an important fungal genus that causes diseases in plants, animals, and humans; however, the occurrence of *Fusarium* species in mangrove soil is largely unexplored. A preliminary study on *Fusarium* species from mangrove soil by Latiffah et al. [13], reported three *Fusarium* spp. In this study, mangrove soil samples were collected from one location, and the three species were identified based solely on morphological characteristics. Thus, it confirmed that mangrove soils harbor *Fusarium* species, and more species might be present. *Fusarium* species residing in mangrove soil are likely saprophytic, as well pathogenic, as many species are facultative parasites. Therefore, mangrove soil could be a reservoir for pathogenic species.

Considering its saprophytic and pathogenic role in plants, animals, and humans, the present study aimed to explore the *Fusarium* species in mangrove soil based on molecular identification and phylogenetic analysis, so as to accurately identify isolates of *Fusarium* at the species level. Additionally, several properties of the mangrove soil were also analyzed.

## 2. Materials and Methods

### 2.1. Isolation and Morphological Identification of Fusarium Isolates

Soil samples were collected from mangrove areas in the northern states of Penang and Kedah, Peninsular Malaysia (Table S1). Samples were collected at a depth of 5.0–15.0 cm from the surface, placed in polythene bags, and transported to the lab for processing. The soil samples were air-dried for 72 h or until fully dried. Approximately 500 g of the soil sample was ground using a mortar and pestle, sieved through a 2 mm strainer, and set aside for later use.

Three isolation methods, namely direct isolation, soil dilution, and debris isolation, as recommended by Leslie and Summerell (2006) [14], were used to isolate *Fusarium* from the mangrove soil samples. The medium used for isolation was peptone-pentachloronitrobenzene-based agar [14].

All isolates successfully isolated from mangrove soils were tentatively identified based on their morphological characteristics as described in The *Fusarium* Laboratory Manual [14].

### 2.2. Molecular Identification and Phylogenetic Analysis

The translation elongation factor-1α (*TEF-1α*) gene was used to confirm the identity of the morphologically identified *Fusarium* isolates as the gene is recommended for identification of *Fusarium* species. In addition, the internal transcribed spacer (ITS) region and *β-tubulin* were included in the phylogenetic analysis depending on the species identified based on the *TEF-1α* gene [14]. ITS region and *β-tubulin* gene were used for phylogenetic analysis of *F. solani* isolates. For the other *Fusarium* spp., *TEF-1α* and *β-tubulin* genes were used to perform phylogenetic analysis.

#### 2.2.1. DNA Extraction

Fungal isolates were cultured in 50 mL potato dextrose broth and incubated for 7 days at 27 ± 2 °C. The mycelium was harvested, dried on a double-layered sterile filter paper, and kept in a freezer overnight. Then, it was lyophilized at −40 °C for 48 h using a freeze dryer (Triad^TM^ Freeze Dry, Labconco, MO, USA). The dried mycelia were ground with liquid nitrogen to a fine powder using a sterile mortar and pestle. DNA was extracted from 0.24 to 0.26 g of the mycelia fine powder using the DNeasy Plant Mini Kit (Qiagen, Hilden, Germany) according to the manufacturer’s protocols.

#### 2.2.2. PCR Amplification

PCR amplification was carried out in a DNA Engine^TM^ Peltier Thermal Cycler Model PTC-100 (MJ Research Inc., Watertown, MA, USA). *TEF-1α* was amplified using primers EF1 (ATG GGT AAG GAR GAC AAG AC) and EF2 (GGA RGT ACC AGT SAT CAT GTT) as described by O’Donnell et al. [15]. The primers used to amplify *β-tubulin* were BT1 (AAC ATG CGT GAG ATT GTA AGT) and BT2 (TAG TGA CCC TTG GCC CAG TTG), as described by O’Donnell and Cigelnik [16]. PCR amplification of both *TEF-1α* and *β-tubulin* was carried out in a 50 µL reaction containing 4.0 µL buffer (Promega, Madison, WI, USA), 4.0 µL MgCl_2_, 0.5 µL dNTP mix (Promega), 4.0 µL of each primer, 0.125 µL *Taq* polymerase (Promega), and 0.5 µL template DNA.

PCR cycle for *TEF-1α* amplification was as follows: initial denaturation at 94 °C for 85 s followed by 35 cycles of denaturation at 95 °C for 35 s, annealing at 59 °C for 55 s, extension at 72 °C for 90 s, and a final extension at 72 °C for 10 min. The PCR cycle for *β-tubulin* amplification was as follows: initial denaturation at 94 °C for 1 min, followed by 30 cycles at 94 °C for 30 s, annealing at 58 °C for 30 s, extension at 72 °C for 1 min, and final extension at 72 °C for 5 min.

The ITS region was amplified using the primers ITS5 (GGA AGT AAA AGT CGT AAC AAG G) and NL4 (GGTC CGT GTT TCA AGA CGG) [17]. PCR amplification was carried out in a 50 µL reaction containing 0.5 µL template DNA, 4.0 µL PCR buffer, 4.0 µL MgCl_2_, 2.0 µL each of forward and reverse primers, 0.5 µL dNTPs mix (Promega), and 0.125 µL of *Taq* polymerase (Promega). The PCR cycle for amplification of the ITS region was as follows: initial denaturation at 95 °C for 2 min, followed by 30 cycles of denaturation at 95 °C for 1 min, annealing at 50 °C for 30 s, extension at 72 °C for 2 min, and final extension at 72 °C for 10 min.

Agarose gel electrophoresis (1.5%) was used to detect genomic DNA and amplified PCR products of *TEF-1α*, *β-tubulin*, and ITS region. Electrophoresis was performed at 70 V and 400 mA for 80 min with 1X TBE buffer. The agarose gel was loaded with 4 µL genomic DNA or PCR product and 1 µL of loading dye (Fermentas, Vilnius, Lithuania). The obtained bands were evaluated using 1 kb and 100 bp DNA markers (Fermentas). After electrophoresis, the gel was viewed using Bio-RAD Molecular Imager^®^ Gel DocTMXr system with Discovery Series TM Quantity One^®^ 1-D Analysis software Version 4.6.5. (Bio-RAD, Hercules, CA, USA). The PCR products were sent to a service provider for DNA sequencing.

#### 2.2.3. Sequence and Phylogenetic Analyses

All sequences were aligned using ClustalW included in the Molecular Evolution Genetic Analysis (MEGA5) software [18]. The consensus sequences obtained from the forward and reverse sequences were compared with other sequences in the GenBank and Fusarium-ID databases. BLAST search was performed in both databases to obtain the identity of the isolates.

Eight *Fusarium* species were included in the phylogenetic analysis to represent other known clades and *Fusarium* spp. as a reference (Table 1).

Phylogenetic analysis of *F. solani* was performed separately, as the isolates were more prevalent than the other *Fusarium* species. For phylogenetic analysis of *F. solani* isolates, other sequences representing known FSSC clades were also included (Table 2).

Multiple sequence analysis for phylogenetic analysis was also performed using MEGA 5 software. Phylogenetic analysis was carried out using the Maximum Likelihood (ML) method, as the ML method deduces an evolutionary tree by maximizing the probability of observing the data. To generate the ML tree, the complete deletion option, Nearest-Neighbor-interchange (NNI), and 1000 bootstrap replications were applied, and the tree was inferred by an NJ algorithm using a matrix of pair distances estimated with the Kimura model.

### 2.3. Physical and Chemical Analyses of Mangrove Soil

Several parameters of the mangrove soil samples, such as moisture, texture, pH, salinity, carbon content, and nitrogen content, were analyzed.

#### 2.3.1. Soil Moisture

The oven-dry method was used to calculate soil moisture. The moisture content in weight percent was derived using the following formula (1) [24]:

Moisture (wt %) = (wt of wet soil + tare) − (wt of day soil + tare)/(wt of dry soil + tare) − (tare) × 100(1)

#### 2.3.2. Soil Texture

The pipette method [25] was used to determine the soil texture. The texture of the soil sample and percentages of clay, sand, and silt were estimated using the United States Department of Agriculture (USDA) soil textural triangle.

#### 2.3.3. Soil pH

Soil pH was measured for a 1:2.5 soil/water suspension [24] using a pH meter (Mettler–Toledo 320, Greifensee, Switzerland).

#### 2.3.4. Soil Salinity

Soil salinity was measured in a suspension of the soil in water (1:2) using an electric conductivity). Salinity readings expressed in terms of ppt.

#### 2.3.5. Carbon Content

The carbon content of the mangrove soil samples was determined using the dichromate oxidation method or the Walkley–Black method [26]. The carbon content was then calculated using the following formula (Rowell, 1994):

(i) Oxidation carbon 75% = volume blank-volume titrate × 0.003 × 100/weight of sample

(ii) Total carbon (%) = Oxidizable carbon (75%) × 100/75

The total amount of carbon was converted using a conversion factor of 1.72 to organic matter by using the following formula: Organic matter (%) = Total carbon × 1.72.

#### 2.3.6. Nitrogen Content

The Kjeldahl method was used to determine the nitrogen content [24] of the mangrove soil samples. The amount of nitrogen was determined using the following formula (2):

Total nitrogen = volume of acid used in the titration process (mL) × 100/weight(2)

### 2.4. Statistical Analyses

All data were analyzed using Statistical Package for the Social Sciences (SPSS) software. One-way ANOVA was applied to compare soil properties at different sampling locations. Duncan’s multiple-range test was used to compare different properties of the mangrove soil samples. This analysis was based on the comparison of three or more means that differ significantly in an analysis of variance. The T test was used to compare mangrove soil sample properties between soil samples from sampling sites in the states of Penang and Kedah.

## 3. Results

### 3.1. Fusarium Isolates

Debris isolation yielded the highest number of *Fusarium* isolates (51%), followed by direct isolation (48%) and soil dilution plate isolation (1%). A total of 124 isolates of *Fusarium* were recovered and tentatively identified as *F. solani* (*n* = 77), *F. proliferatum* (*n* = 14), *F. verticillioides* (*n* = 12), *F. incarnatum* (*n* = 10), *F. oxysporum* (*n* = 3), *F. camptoceras* (*n* = 2), *F. lateritium* (*n* = 4), and *F. decemcellulare* (*n* = 2).

### 3.2. PCR Amplification and Sequence Analysis of TEF-1α Gene

Successful amplification of *TEF-1α* using EF1 and EF2 primer pairs was confirmed by the presence of a single 750 bp band using agarose gel electrophoresis.

Based on a BLAST search of *TEF-1α* sequences, the isolates were molecularly identified as FSSC (*n* = 77), *F. verticillioides* (*n* = 20), *F. incarnatum* (*n* = 10), *F. proliferatum* (*n* = 7), *F. lateritium* (*n* = 4), *F. oxysporum* (*n* = 3), *F. rigidiuscula* (*n* = 2), and *F. camptoceras* (*n* = 1). All the sequences were deposited in the Genbank and the accession numbers are shown in Supplementary Table S2. BLAST results based on the Genbank and Fusarium-ID databases are shown in Table S3 (FSSC) and Table S4 (other *Fusarium* species).

### 3.3. PCR Amplification and Sequence Analysis of β -Tubulin Gene and ITS Region

A single band of 900 bp observed after amplification of ITS region in the FSSC isolates (*n* = 77). An approximately 650 bp fragment of *β -tubulin* was amplified using BT1 and BT2 primers in the other *Fusarium* species. BLAST search results of *β -tubulin* gene and ITS sequences are shown in Table S3 and accession numbers in Table S2.

### 3.4. Phylogenetic Analysis of FSSC Isolates Based on Combined TEF-1α and ITS Sequences

**Figure 1:**
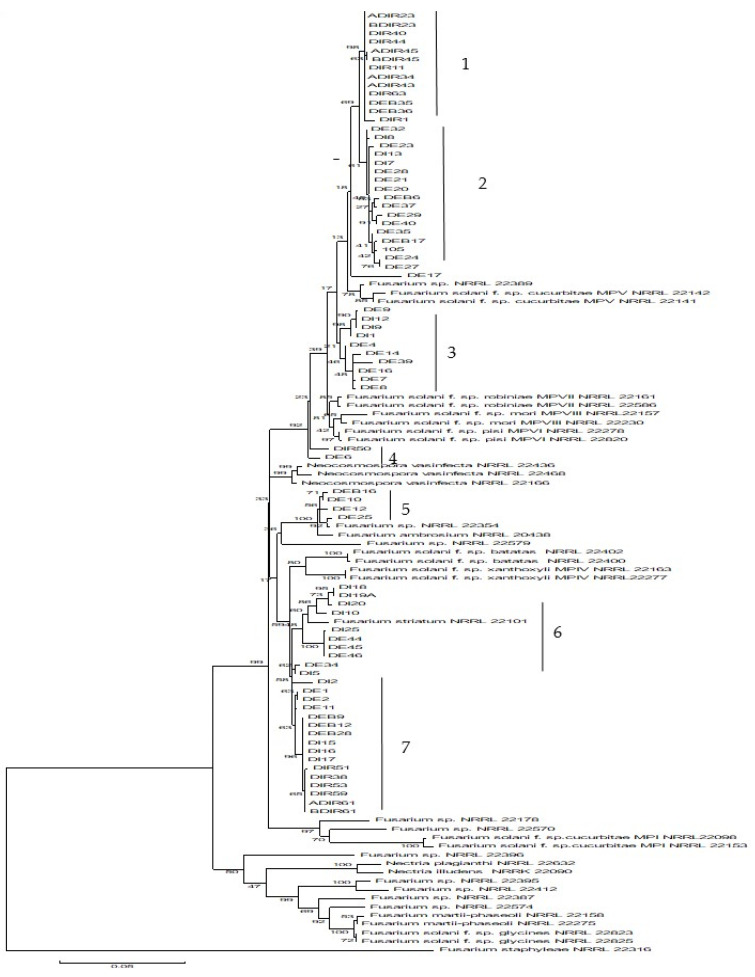
Maximum likelihood tree of 77 isolates of *F. solani* species complex (FSSC) isolated from mangrove soil samples and strains from Genbank based on combined sequences of ITS and TEF-α using Kimura 2+ I parameter method. FSSC isolates are grouped in seven clades (1–7).

The ML tree generated based on combined TEF-1α and ITS sequences revealed diverse phylogenetic affinities among FSSC isolates from mangrove soils. The isolates were grouped into seven clades 1, 2, 3, 4, 5, 6, and 7 (Figure 1). Only isolates in clades 5 and 6 were grouped with *F. solani* reference strains described by O’Donnell [19]. Other FSSC isolates (Clades 1, 2, 3, 4, and 7) from mangrove soil did not group with any of the reference strains described by O’Donnell [19].

Four FSSC isolates (DEB16, DE10, DE12, and DE25) were grouped with two reference strains, *Fusarium* sp. 22354 and *F. ambrosium* NRRL20438 in clade 5. Four other isolates (DI20, DI10, DI18, and DI19A) in clade 6 were clustered with the reference strain of *F. striatum* NRRL22101 (Panama) member of clade 3 of *F. solani*–*Nectria haematococca* clades and had 98%–99% similarity with *N. ipomoeae.*

### 3.5. Phylogenetic Analysis of Fusarium Species Based on Combined TEF-1α and β -Tubulin Sequences

**Figure 2:**
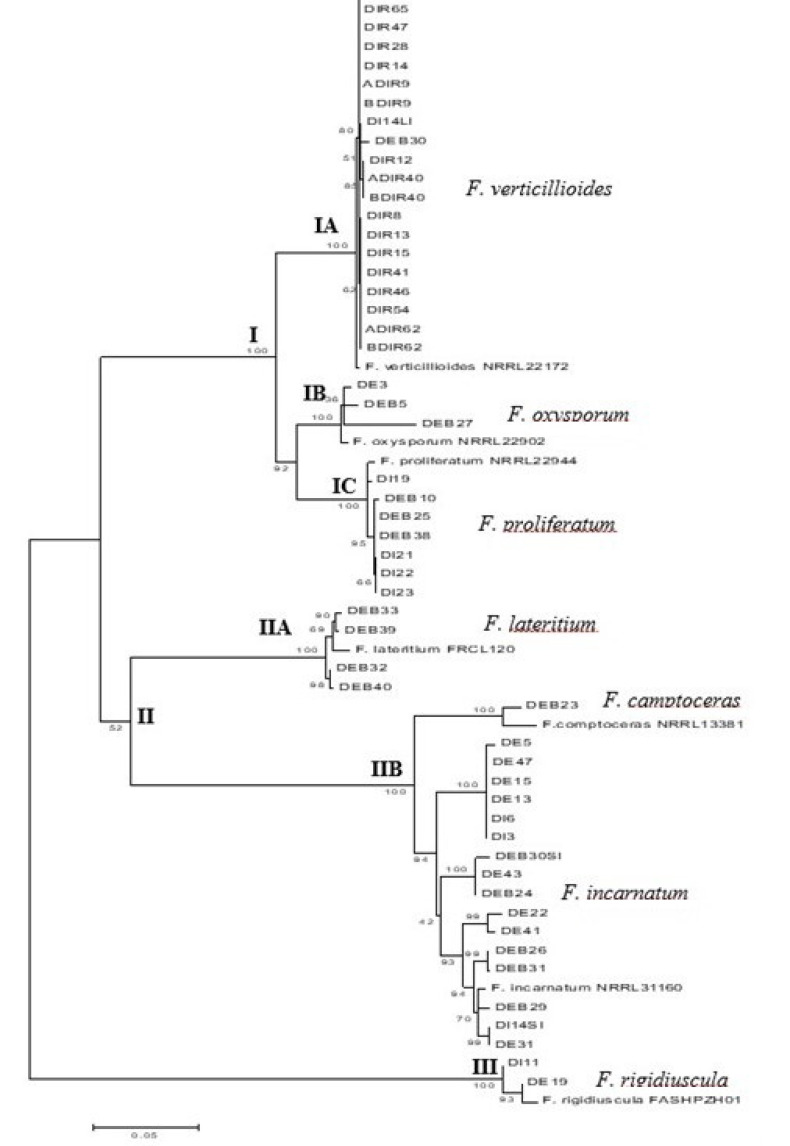
Maximum likelihood tree of *Fusarium* species from mangrove soil samples based on *β-tubulin* and *TEF-α* sequences. The percentage of bootstrap value was higher than 50 % are shown next to the branches. The *Fusarium* spp. are grouped in three main clades (I, II and III) with several sub-clades (IA, IB, IC, IIA and IIB).

In the ML tree analysis, isolates belonging to the same species were grouped in the same clade (Figure 2). The tree was divided into three main clades, I, II, and III. Main clade I consisted of *F. verticillioides* (sub-clade IA), *F. oxysporum* (sub-clade IB), and *F. proliferatum* (sub-clade IC) isolates; clade II comprised *F. lateritium* (sub-clade IIA), *F. camptoceras,* and *F. incarnatum* (sub-clade IIB); and clade III consisted of only *F. rigidiuscula* (Figure 2).

Therefore, based on molecular identification, the most common species recovered from the mangrove soil samples was FSSC (*n* = 77)*,* followed by *F. verticillioides* (*n* = 20), *F. incarnatum* (*n* = 10), *F. proliferatum* (*n* = 7), *F. lateritium* (*n* = 4), *F. oxysporum* (*n* = 3), *F. rigidiuscula* (*n* = 2), *F. chlamydosporum* (*n* = 1)*,* and *F. camptoceras* (*n* = 1).

### 3.6. Soil Analysis

The results of mangrove soil physical and chemical analyses, *Fusarium* species identified at each sampling site, and the total number of isolates of each species are presented in Table 3. Seven *Fusarium* species were recovered from PB3 in Pulau Burung, Penang, namely FSSC, *F. verticillioides*, *F. incarnatum*, *F. proliferatum, F. oxysporum, F. chlamydosporum,* and *F. camptoceras*. The highest number of *Fusarium* isolates was recovered from Pantai Acheh, Penang, with 48 isolates.

#### 3.6.1. Soil Moisture

Soil moisture content significantly differed between the sampling locations (*p* < 0.05). Soil moisture content was 2.59%–3.7% in Penang (PG and PB) and 1.6%–4.8% in Kedah (KPM, KBL, S1SM, S2SM, and KSG). Moisture content at KBL was significantly different from that at the other sampling locations.

There was no significant difference in soil moisture content between sampling locations in Penang and Kedah. The highest number of isolates (*n* = 40) occurred at PG with a moisture content of 3.07%. Seven *Fusarium* species were identified at PB3 with a moisture content of 3.3% (Table 3).

#### 3.6.2. Soil Texture

The mangrove soil samples from Penang were sandy loam soil, while those from Kedah varied from sandy loam and silt loam to sandy clay loam (Table 3).

#### 3.6.3. Soil pH

The pH value of the mangrove soil samples ranged from neutral to acidic, and significant differences were observed between sampling locations (*p* < 0.001). At two sampling sites, PG and PB, in Penang the soil pH was neutral to slightly acidic (pH 6.22–7.5), while at five sampling sites (KPM, KBL, S2SM S1SM, and KSG) in Kedah it was acidic (pH 5.02–6.6) (Table 3).

There was a significant difference (*p* < 0.05) in pH between Penang and Kedah sampling sites. Seven *Fusarium* species were obtained from PB3 in Penang with a soil pH of 7.02.

#### 3.6.4. Soil Salinity

Analysis of soil salinity showed a significant difference between sampling locations (*p* < 0.05). The soil salinity differences were within moderate values ranging from 10.02 to 18.0 ppt at all sampling locations, except at one site (PG3) with higher salinity of 21.36 ppt where the highest number of *Fusarium* isolates recovered (*n* = 40). Seven *Fusarium* species were recovered at PB3 with a salinity of 19.08 ppt.

#### 3.6.5. Carbon Content

Significant variations in soil carbon content were detected between the sampling locations (*p* < 0.001). The carbon content in two sites, PG and PB in Penang ranged from 49 mg/g to 138 mg/g, while that in KPM, KBL, S1SM, S2SM, and KSG in Kedah ranged from 15.75 mg/g to 58.0 mg/g (Table 3). The carbon content between sampling sites in Penang and Kedah showed a significant difference (*p* < 0.05).

Seven *Fusarium* species were found at PB3, however, the highest number of isolates (*n* = 48) was obtained from PG (Table 3).

#### 3.6.6. Nitrogen Content

The nitrogen content in the soil samples did not significantly differ among the sampling locations. The highest nitrogen content of 4.0 mg/g was recorded at the PB1 in Penang and two isolates of *F. lateritium* were recovered there (Table 3).

## 4. Discussion

Most of the *Fusarium* species reported in this study were isolated using the debris isolation method, which yielded the highest number of isolates from the mangrove soil samples. Debris in mangrove soil consists of plant roots, dried leaves, stems, and branches, which often serve as habitats for saprophytic and parasitic fungi that can exist as discrete propagules, such as chlamydospores and resistant conidia [2]. Most *Fusarium* species produce chlamydospores, resistant conidia, and resistant hyphae in the soil, which germinate under suitable conditions, such as nutrient availability [27]. Additionally, carbon-rich soils can harbor adherent conidia and fungal fragments for extended periods of time.

Phylogenetic analysis of FSSC isolates from mangrove soil suggested that several isolates might represent new phylogenetic species or distinct lineages of *F. solani* residing in mangrove soil as isolates in clades 1, 2, 3, 4, and 7 did not group with any clade or lineage described by O’Donnell [19]. New phylogenetic lineages of *F. solani* from native soils have been reported by Nalim et al. [28], who collected *F. solani* isolates from the soil of primary forests in Sri Lanka and tropical Australia. The *F. solani* isolates formed new novel clades within clade 3 [19]. New phylogenetic clades or lineages were previously reported by Bogale et al. [29], Nalim et al. [28], and Chehri [30] indicating that *F. solani* contains numerous phylogenetic lineages. The numerous phylogenetic lineages of *F. solani* might be associated with the widespread occurrence and distribution of FFSC, of which this species has a cosmopolitan distribution and is a common soil inhabitant.

Phylogenetic analysis of FSSC isolates also suggested four FSSC isolates from mangrove soil were related to *N. ipomoeae*, which is a well-known saprophyte and pathogen causing stem rot in eggplant and sweet potato. These FSSC isolates were clustered with the reference strain of *F. striatum* NRRL 22101 (Panama) member of clade 3 described by O’Donnell (2000). *F. striatum* is an anamorph of *Haematonectria ipomoeae* (Halst) [31] and is synonymous with *Nectria ipomoeae* [32].

FSSC isolates are common soil inhabitant and have been isolated from a variety of soil types, including subtropical and semi-arid grassland soil [33], saline soil [34], peat soil [4], native soils in Sri Lanka and tropical Australia [28], cultivated soil [35], and agriculture soil [29].

*F. verticillioides* and *F. proliferatum* are closely related, as both are members of the FFSC, whereas *F. oxysporum* is considered a sister group of the FFSC [16,19]. These three species are widely distributed and have been recovered from various hosts or substrates. Both *F. verticillioides* and *F. proliferatum* are well-known plant pathogens associated with corn ear rot. Furthermore, there are several reports on *F. verticillioides* and *F. proliferatum* isolated from non-agricultural soils [13,36,37,38]. The occurrence of both *F. verticillioides* and *F. proliferatum* in mangrove soil might be attributed to the dispersal of infected plant materials via wind.

*F. oxysporum* is a common soil saprophyte that has been isolated from agricultural soil [14], cultivated, and temperate soils [39,40]. Similar to *F. verticillioides* and *F. proliferatum,* the dispersal of *F. oxysporum* to mangrove areas might be through infected planting material; however, this species produces chlamydospores and conidia to ensure long-term survival in the soil and plant debris, which can be dispersed via wind, soils, and seeds [41].

*F. incarnatum* and *F. camptoceras* isolates are part of the *Fusarium incarnatum-equiseti* species complex (FIESC); however, based on a study by Xia et al. [42], *F. camptoceras* formed three lineages that are distinct from FIESC. *F. camptoceras* was reported from fynbos soils [43] and the distribution of this species is restricted to tropical and sub-tropical areas [14].

Similar to FSSC isolates, *F. incarnatum* is also widespread, commonly found in the tropics and subtropics, but has also been reported in the Mediterranean and in temperate areas [14]. It is a common soil inhabitant that has been recovered from peat soil [44], sandy soil [45], and Arctic soil [6].

*F. lateritium* occurs in soil worldwide [14] and has been reported in soil samples from different altitudes in the Transkei, Southern Africa [46], soil planted with millet and sorghum from Lesotho, Nigeria, and Zimbabwe [47], and from and in desert zones of Dead Sea Valley [48].

As *F. rigidiuscula* (syn *F. decemcellulare*) is frequently found in tropical regions, it is not surprising that this species was isolated from mangrove soil. Most reports on *F. rigidiuscula* are associated with plant diseases, including the death and gall in mango trees and bark canker of hardwood trees. *F. decemcellulare* was also found in non-agricultural garden soil samples from Hormozgan province, Iran [49].

The number of *Fusarium* isolates and species recovered from each sampling location in the present study varied. This variation might be related to the soil physical and chemical properties, such as soil moisture, soil pH, carbon and nitrogen content, and soil texture, as these factors influence the distribution of fungal diversity in the soil.

The highest number of *Fusarium* isolates identified were in sandy loam soils. Sandy loam soil has high moisture content and aeration, which are suitable for the growth of microorganisms. Soil texture is one of the most important factors influencing the structure of microbial communities, including fungi. Kara and Bolat [50] studied the effect of soil compaction on microfungal community structure and reported that the frequency of the genus *Fusarium* was greatly increased in sandy loam soil.

Panda et al. [51] reported the influence of soil total carbon, total nitrogen, pH, temperature, moisture, and surface vegetation on soil microflora in a coastal, sandy belt of India, and concluded that carbon content positively affects fungal populations, including *Fusarium* species. Soil carbon content is a vital source of energy for the inhabitant microorganisms that inhabit the soil. Organic matter serves two functions for microorganisms in soils, providing energy for growth and supplying carbon for the formation of new cells [52]. The results of the present study were similar to those reported by Swer et al. [53], who stated that the fungal population is positively influenced by organic carbon, which can play an essential role in growth, sporulation, and diversity. The carbon content at the sampling sites in Kedah was significantly lower than that in Penang. The number of *Fusarium* isolates recovered in all sampling locations in Kedah was less than that in Penang, wherein *F. solani* and *F. incarnatum* were the prevalent species. These two species are well known soil fungi and can be found in various soil environments, such as in tropical and subtropical regions [14] and are capable of tolerating unsuitable conditions as they produce chlamydospores that can withstand extreme conditions [2] such as less nutrition, desiccation, anaerobic conditions, and high temperature. *Fusarium* isolates were not recovered at several sampling sites (PB1, KBL2, and SISM2), which had high carbon content, may be due to competition with other microorganisms. According to Leslie and Summerell [14] the population structure of *Fusarium* spp. in non-cultivated and cultivated soils depends on several factors, including the capacity to colonize different plant hosts and to compete saprophytically with other microbial communities.

The nitrogen content in all mangrove soil samples, did not differ significantly. It is possible that high nitrogen content in the soil increases fungal sporulation and hyphal growth rate [54], which contributes to the occurrence of *Fusarium* isolates in the mangrove soils. Joshi and Chauhan [55] studied the distribution of microfungi in various soil types from Chambal ravines, India and reported a significant positive influence of nitrogen content on fungal populations, including *Fusarium*.

Soil pH strongly influences the availability of carbon and nutrients as well as the solubility of metals. Fungi, including *Fusarium*, exhibit a wider pH range from 2 to 10 for optimal growth [56]. A significant difference in pH, ranging from acidic to neutral, was detected between the sampling locations. Several studies have shown that the pH of mangrove soils can range from acidic to alkaline. Gonzalez-Acosta et al. [57] and Thatoi et al. [58] reported nominal alkalinity in the Mexican mangrove forest and slight alkalinity in Bhitarkanika Odisha mangrove sediment samples, respectively. Notably, microbial groups differ in their responses to soil pH, and a few studies have reported the impact of soil pH on fungal communities and their occurrences. Wheeler et al. [59] and Nevarez et al. [60] pointed out that fungal species typically have a wide range of optimum pH, often ranging from pH 5 to 9, without significant inhibition of their growth. Nazim et al. [61] stated that fungi were capable of enduring acidic conditions and were equally abundant in acidic and neutral soils, which was similar to the present study findings. Maina et al. [62] studied the effect of pH on different types of soils in Taita Taveta District, Kenya, focusing on the distribution and diversity of *Fusarium* species, and concluded that there was a positive effect of pH levels and the occurrence and abundance of *Fusarium*.

Soil salinity was found to be significantly different among sampling locations, but remained within a moderate range of 5–18 ppt. The two most prevalent *Fusarium* species, FSSC and *F. verticillioides*, were recovered from a sampling site with a slightly high salinity of 21.36 ppt suggesting that the effect of salinity may have altered the competitive ability of other microbes in the soil. Mandeel [34] studied the biodiversity of the genus *Fusarium* in saline soil habitats in Bahrain and identified five *Fusarium* species, *F. solani*, *F. oxysporum*, *F. chlamydosporum*, *F. longipes,* and *F. compactum*, of which *F. solani* was dominant and could tolerate high salinity conditions.

Soil microbial biomass can be limited by soil moisture under both dry and wet conditions. Mandeel [34] reported that *Fusarium* species recovered from saline habitats in Bahrain was influenced by soil moisture. Banakar et al. [63] concluded that soil moisture negatively affected soil fungi populations, including *Fusarium*, from dry deciduous forest. Oritsejafor [64] reported that the highest survival of *F. oxysporum* as a pathogen was recorded at the lowest level of soil moisture tested (15%), which indicates that *Fusarium* spp. are strongly aerobic, and that *Fusarium* population can be reduced by maintaining the soil in a saturated condition. *Fusarium* could not be isolated from soil submerged in water for 12 weeks.

*Fusarium* spp. in mangrove soil are likely saprophytes involved in many aspects of degradation of organic materials, nutrient cycling, and energy flow in mangrove ecosystems. Among the *Fusarium* species identified, FSSC, *F. oxysporum, F. verticillioides*, and *F. proliferatum* are well-known plant pathogens that cause vascular wilt, stem and root rot, blight, and damping-off in various agricultural crops. Moreover, *Fusarium* is capable of infecting plants as well as humans, an event known as trans-kingdom pathogenicity [65]. *Fusarium* species also cause opportunistic infections in humans, including superficial (keratitis and onychomycosis), locally invasive, or disseminated infections, particularly in immunocompromised individuals [66]. Most of the reported opportunistic human pathogens are FSSC, *F. oxysporum*, and occasionally *F. incarnatum*, *F. proliferatum*, *F. verticillioides,* and *F. chlamydosporum* [66,67]. Thus, the present study suggests that mangrove soil can be a reservoir for plant and human pathogens, or sources of inoculum for plant disease and human pathogens.

To summarize, *F. solani* species complex (FSSC), *F. oxysporum*, and *F. incarnatum* are common soil inhabitants and are regarded as cosmopolitan species. *F. verticillioides, F. lateritium*, and *F. proliferatum* are also widely distributed worldwide. These species have the ability to occupy and adapt to a wide range of ecological conditions that contribute to their widespread distribution [68]. According to Smith [69], widespread distribution of *Fusarium* spp. might be related to their ability to breakdown and digest various complex carbohydrates and proteins, tolerate adverse climatic conditions, and withstand rather high levels of toxic substances, such as ammonia, antibiotics, and fungicides, which can eradicate other microbes.

## 5. Conclusions

The present study identified several *Fusarium* species in mangrove soil from northern region of Peninsular Malaysia. Based on analyses of soil physico-chemical properties, carbon content, pH, soil moisture, and salinity, varied between the sampling locations, but nitrogen content did not show any significant difference between all locations and between the sampling sites from states of Penang and Kedah. The present study found that regardless of the soil physical and chemical properties, mangrove soils harbor various species of *Fusarium*, with FSSC being the most prevalent. Some species, such as FSSC, *F. oxysporum*, *F. incarnatum*, *F. verticillioides, F. lateritium*, and *F. proliferatum* are distributed worldwide and occur in various hosts and substrates. The various *Fusarium* species residing in mangrove soils are likely saprophytes and may have an indirect effect on plants and humans. The species recovered from mangrove soils have the potential for causing plant diseases under suitable environmental conditions as well as becoming opportunistic human pathogens, thereby posing additional threat to immunocompromised individuals. The present results also indicate that mangrove soil may be a source of inoculum for plant and human pathogenic *Fusarium* species. Moreover, the diverse species of *Fusarium* recovered from mangrove soil adds to the available data on the diversity of the genus *Fusarium*, particularly in non-agricultural environments.

## Figures and Tables

**Table 1:** *Fusarium* species isolates included in phylogenetic analysis.

Species/Code	Accession No.	Geographic Origin	References
*F. verticillioides*	NRRL22172	Germany	O’Donnell et al. [19]
*F. oxysporum*	NRRL22902	USA	O’Donnell et al. [19]
*F. proliferatum*	NRRL22944	Germany	O’Donnell et al. [19]
*F. camptoceras*	NRRL13381	-	Proctor et al. [20]
*F. incarnatum*	NRRL31160	-	Proctor et al. [20]
*F. rigidiuscula*	FASHPZH01	-	Qi et al. [21]
*F. lateritium*	FRCL120	Africa	Geiser et al. [22]
*F. chlamydosporum*	NRRL 52797	India	O’Donnell et al. [23]

**Table 2:** *Fusarium solani* strains included in phylogenetic analysis.

Code	Accession Number	*Fusarium solani*	Geographic Origin
NRRL22389	AF178340	*Fusarium* sp.	Maryland, USA
NRRL22142	AF178347	*Fusarium solani f.* sp. *cucurbitae* MPV	California, USA
NRRL 22141	AF178329	*Fusarium solani f.* sp. *cucurbitae* MPV	New Zealand
NRRL22161	AF178330	*Fusarium solani f.* sp. *robiniae* MPVII	Japan
NRRL22586	AF178353	*Fusarium solani f.* sp. *robiniae* MPVII	Virginia, USA
NRRL22157	AF178359	*Fusarium solani f.* sp. *mori* MPVIII	Japan
NRRL22230	AF178358	*Fusarium solani f.* sp. *mori* MPVIII	Japan
NRRL22278	AF178337	*Fusarium solani f.* sp. *pisi* MPVI	-
NRRL22820	AF178355	*Fusarium solani f.* sp. *pisi* MPVI	Indiana, USA
NRRL22579	AF178352	*Fusarium* sp.	Indonesia
NRRL22163	AF178382	*Fusarium solani f.* sp. *xanthoxyli* MPIV	Japan
NRRL22277	AF178336	*Fusarium solani f.* sp. *xanthoxyli* MPIV	Japan
NRRL22402	AF178344	*Fusarium solani f.* sp. *batatas*	North Carolina, USA
NRRL22400	AF178343	*Fusarium solani f.* sp. *batatas*	North Carolina, USA
NRRL22101	AF178333	*Fusarium striatum*	Panama
NRRL22166	AF178350	*Neocosmospora vasinfecta*	Illinois, USA
NRRL22436	AF178348	*Neocosmospora vasinfecta*	South Africa
NRRL22468	AF178349	*Neocosmospora vasinfecta*	Guinea
NRRL22178	AF178334	*Fusarium* sp.	Venezuela
NRRL22570	AF178360	*Fusarium* sp.	Brazil
NRRL22098	AF178327	*Fusarium solani f. * sp. * cucurbitae* MPI	-
NRRL22153	AF178346	*Fusarium solani f. * sp. * cucurbitae* MPI	-
NRRL20438	AF178332	*Fusarium ambrosium*	India
NRRL22354	AF178338	*Fusarium* sp.	French Guiana
NRRL22396	AF178342	*Fusarium* sp.	French Guiana
NRRL22395	AF178341	*Fusarium* sp.	Venezuela
NRRL22412	AF178351	*Fusarium* sp.	French Guiana
NRRL22387	AF178339	*Fusarium* sp.	French Guiana
NRRL22574	AF178345	*Fusarium* sp.	Guatemala
NRRL22158	AF178331	*Fusarium martii-phaseoli*	New York, USA
NRRL22275	AF178335	*Fusarium martii-phaseoli*	-
NRRL22823	AF178356	*Fusarium solani f.* sp. *glycines*	Indiana, USA
NRRL22825	AF178357	*Fusarium solani f.* sp. *glycines*	Indiana, USA
NRRL22632	AF178354	*Nectria plagianthi*	New Zealand
NRRL22090	AF178326	*Nectria illudens*	New Zealand
NRRL22316	AF178361	*Fusarium staphyleae*	New Jersey, USA

(O’Donnell 2000).

**Table 3:** Mangrove soil properties and *Fusarium* species isolates.

State (Location)	Sampling Site	Soil Texture	Carbon Content (mg/g)	Nitrogen Content (mg/g)	Soil pH	Soil Moisture (%)	Soil Salinity (ppt)	*Fusarium* Species (Number of Isolates)	Total
**Penang** (Pantai Acheh, Balik Pulau	PG1	Loamy sand	71.8 ± 0.9	2.3 ± 0.1	7.33 ± 0.01	2.59	17.39 ± 4.7	*F. solani* (3) *F. lateritium* (2) *F. proliferatum* (1)	48 (35%)
	PG2	Sandy loam	60.96 ± 1.2	1.8 ± 0	7.53 ± 0.39	3.71	16.49 ± 1.4	*F. solani* (1) *F. longipes* (1)	
	PG3	Sandy loam	49.52 ± 0.6	1.9 ± 0	7.29 ± 0.01	3.07	21.36 ± 0.6	*F. solani* (22) *F. verticillioides* (18)	18 (13%)
**Penang** (Pulau Burung, Seberang Perai) **Pinang** (Pulau Burung, Seberang Perai)	PB1	Sandy loam	138 ± 1.6	4.0±0	6.22±0.19	3.64	16.72 ± 0.5	*F. lateritium* (2)	
	PB2	Sandy loam	84.5 ± 0.3	1.33 ± 0.4	7.14 ± 0.06	2.43	16.24 ± 0.05	*F. oxysporum* (1)	
	PB3	Sandy loam	113 ± 8.6	3.4 ± 0.2	7.02 ± 0.06	3.3	19.08 ± 2.9	*F. solani* (4) *F. verticillioides* (1) *F. incarnatum* (5) *F. proliferatum* (2) *F. oxysporum* (1) *F. chlamydosporum* (1) *F. camptoceras* (1)	
**Kedah** Kampung Pantai Merdeka	KPM1	Sandy loam	31.93 ± 0.7	1.7 ± 0.2	6.61 ± 0.01	3.6 ± 0.5	18.91 ± 4.3	*F. solani* (4)	13 (9%)
	KPM2	Sandy loam	21.03 ± 1.2	1.33 ± 0.1	6.12 ± 0.05	1.83 ± 0	18.31 ± 0.1	*F. solani* (5) *F. incarnatum* (3) *F. oxysporum* (1)	
	KPM3	Loam	19.45 ± 0.8	1.6 ± 0.2	5.97 ± 0.01	2.14 ± 0.3	16.52 ± 0.22	0	
**Kedah** Kampung Batu Lintang	KBL1	Silt loam	35.58 ± 0.8	2.46 ± 0.1	5.72 ± 0.02	4.82 ± 0.4	10.61 ± 0.5	*F. solani* (13) *F. incarnatum* (3) *F. rigidiuscula* (1)	17 (12%)
	KBL2	Sandy loam	47.36 ± 2.7	2.46 ± 0.1	5.4 ± 0.03	3.9 ± 0.2	17.62 ± 0.07	0	
	KBL3	Sandy loam	32.016 ± 1	2.0 ± 0	5.27 ± 0.09	2.77 ± 0.1	13.37 ± 0.02	*F. merismoides* (1)	
**Kedah** Semeling1 Sungai Merbok	S1SM1	Clay loam	28.61 ± 2.5	2.6 ± 0	5.02 ± 0.01	1.62 ± 0.4	11.99 ± 0.6	*F. solani* (1)	2 (1.4%)
	S1SM2	Sandy loam	58. ± 27 ± 1	1.6 ± 0	5.33 ± 0.02	3.08 ± 0.4	13.2 ± 0.22	0	
	S1SM3	Sandy loam	28.86 ± 0.1	1.8±0.1	5.36±0	1.7±0.08	10.25±0.04	*F. rigidiuscula* (1)	
**Kedah** Semeling2 Sungai Merbok	S2SM 1	Sandy clay loam	45.59 ± 0.4	2.1 ± 0.11	5.31 ± 0.01	2.66 ± 0.2	12.78 ± 0.6	*F. solani* (4) *F. incarnatum* (2)	20 (14%)
	S2SM 2	Sandy clay loam	42.24 ± 1.5	2.4 ± 0	5.11 ± 0	2.66 ± 0.2	11.89 ± 0.14	*F. solani* (5) *F. verticillioides* (1) *F. incarnatum* (2)	
	S2SM 3	Sandy loam	27.69 ± 7.5	2 ± 0	5.57 ± 0.03	1.82 ± 0.2	10.02 ± 0.15	*F. solani* (1) *F. longipes* (1)	
**Kedah** Segantang Garam **h** Segantang Garam	KSG1	Loam	20.4 ± 0.13	2.06 ± 0.1	5.98 ± 0.07	4.21 ± 0.1	13.02 ± 0.3	*F. solani* (1)	18 (13%)
	KSG2	Silt loam	42.75 ± 0.7	1.6 ± 0	6.12 ± 0.01	2.94 ± 0.0	11.36 ± 0.8	*F. solani* (10) *F. incarnatum* (1) *F. proliferatum* (2) *F. longipes* (1)	
	KSG3	Silt loam	26.85 ± 0.5	2.06 ± 0.1	6.133 ± 0	4.0±0.7	13.33±0.3	*F. proliferatum* (3)	

## Data Availability

The data presented in this study are available on request from the corresponding author.

## Supplementary Materials

The following are available online https://www.mdpi.com/2076-2607/9/3/497/s1; Table S1. Sampling locations of mangrove soil samples collected in the northern states of Penang and Kedah, Peninsular Malaysia, Table S2. Accession numbers of TEF-1α, ITS and *β*–tubulin sequences of all *Fusarium* species, Table S3. BLAST search results and percentage of similarity of TEF-1α sequences (FFSC and other *Fusarium* species) based on the Genbank and Fusarium-ID databases, Table S4. BLAST search results and percentage of similarity of ITS and *β*–tubulin sequences (FFSC).
